# Effects of Heating Treatment on Functional and Structural Properties of Liquid Whole Egg

**DOI:** 10.3390/foods12071474

**Published:** 2023-03-30

**Authors:** Huiyong Wang, Yanqiu Ma, Yujie Chi

**Affiliations:** College of Food Science, Northeast Agricultural University, Harbin 150030, China; huiyong163@163.com (H.W.); yqma2019@neau.edu.cn (Y.M.)

**Keywords:** liquid whole egg, heating treatment conditions, emulsification, foaming activity, rheological properties

## Abstract

Liquid whole egg (LWE) products have many advantages such as convenient transportation, easy production and are safe. However, LWE has a short shelf life and high thermal sensitivity, so suitable heating treatment is the key to the production of LWE products. The aim of this study is to investigate the effects of heating treatments conditions (at 55–67 °C for 0–10 min) on the emulsification, foaming activity and rheological properties of LWE. The results indicated that the emulsifying activity of LWE had no significant change after 55–64 °C heating treatment, while it decreased significantly after heating treatment at 67 °C. The foaming property of LWE increased significantly after 55 °C to the 64 °C heating treatment; while the foaming property showed a downward trend with the increase in heat treatment temperature, it can significantly improve the foam stability of LWE. The heating treatment thoroughly changed the molecular weight distribution of LWE protein, thus promoted the protein surface hydrophobicity, hydrophobicity activity and rheological properties. The heating treatment at 61 °C for 6 min had a better effect on the functional properties than that of the other heating groups. In addition, the results of this study provide the change in rules of LWE under different heating treatment conditions and provide theoretical guidance for the production and processing of LWE.

## 1. Introduction

Eggs, a type of economical food with high nutritional value, are widely used for food processing [1]. However, the simple white or yolk in eggs cannot fulfill the individual demands of some special consumers, such as athletes, pregnant women, infants and obese people. Now, liquid whole egg (LWE) products have attracted wide attention due to its nutritional value and processing characteristics [1,2]. LWE products are made from fresh eggs after shelling, mixing egg white and yolk in proportion, sterilization, packaging and other processing processes. Currently, LWE products account for 35–45% of the total egg production in the United States, Japan, the European Union and other countries [3]. However, LWE products may be contaminated by spoilage and pathogenic microorganisms such as *Salmonella* during processing, and the egg liquid is rich in protein and other nutrients suitable for microbial growth and reproduction, resulting in a short shelf life of the egg liquid, which greatly limits the application of LWE [4].

Currently, thermal processing, such as cooking, pasteurization and sterilization, is widely used to preserve and extend the shelf life of liquid food due to its excellent sterilization performance and role of improving food flavor [1,5]. The microbiological safety of LWE products is also mainly guaranteed by heating treatment in food. For example, the USDA requires that LWE is at least heated at 60 °C for no less than 3.5 min, but in the United Kingdom, the recommendations are to heat treatment to at least 64 °C for 2.5 min [2,6,7]. However, egg protein is sensitive to temperatures and is easy to solidify and denature in case of heating treatment, which seriously affects the quality of liquid egg and products. Therefore, it is particularly important to take appropriate heating treatment conditions to ensure LWE product quality.

A previous study has shown that the structural properties of hen egg white protein are highly dependent on the heating temperature [8]. In addition, the structural changes of protein in eggs during heat treatment further lead to the differences in functional properties. Tang et al. compared the edible quality and antioxidant activity of liquid egg after frying, microwaving, steaming and baking [7]. They showed that heat treatment significantly improved the texture and microstructure of eggs and enhanced their antioxidant activity. Nolasco et al. [9] showed that the gastrointestinal digestibility of egg and bioaccessibility of different bioactive compounds are affected by cooking methods in different temperatures. Uysal et al. [1] used the capillary electrophoresis techniques and ultraviolet–visible (UV–VIS) spectroscopy to analyze the effect of the treatment parameters (temperature and time) on egg proteins and showed that the solubility of protein is highly related to the heat treatment parameters. In addition, heating treatment has been proven to improve the functional properties of bioactive substances such as protein and lecithin in eggs [10,11]. At present, most studies focus on egg white or egg yolk alone, while few studies have been conducted on the effect of heating treatment conditions on the functional properties of the complex LWE system.

The aim of this study is to investigate the effect of different heating treatment temperatures and time on the emulsification, foaming activity and rheological properties of LWE. The study is intended to provide the theoretical basis for the development of LWE and its products.

## 2. Materials and Methods

### 2.1. Materials

Eggs were purchased from a supermarket. Sodium dodecyl sulfonate (SDS), 1-phenylaminonaphthalene-8-sulfonic acid (ANS) and 5,5 ‘dithiobis (2-nitrobenzoic acid) (DTNB) were obtained from Sigma-Aldrich (St. Louis, MO, USA). Other chemicals were of analytical grade.

### 2.2. Methods

#### 2.2.1. LWE Samples Preparation

According to the methods used by Uysal et al. [1], eggs were manually broken and homogenized (IKA T-18 Ultra Turrax Digital Homogenizer, Germany) at 6500 rpm for 3 min. Then, a 500 mL LWE sample was taken and placed in a beaker, and preheated in a constant-temperature water bath at the set temperature. The heating temperature was 55, 58, 61, 64 and 67 °C, and the heating time was 0, 2, 4, 6, 8 and 10 min, respectively. A camera was used to photograph the apparent morphology of LWE sample.

#### 2.2.2. Solubility Determination

Fresh LWE and LWE with different sterilization treatments were centrifuged at 4 °C and 10,000× *g* for 10 min. The protein content in the supernatant was determined by the Coomassie Brilliant Blue method [12], and the total protein content was determined by the Kjeldahl method [13]. The protein solubility was calculated by the following formula:

(1) $$Solubility\left(\%\right)=\frac{protein\;contet\;in\;suoernatant}{total\;protein\;content}\times 100$$

#### 2.2.3. Determination of Emulsifying Properties

The phosphate-buffered solution (pH = 7.0, 0.1 mol/L) was used to dilute the LWE sample into a solution with a protein content of 0.2% (*w*/*v*), 30 mL of the protein solution was taken and 10 mL of soybean oil was added and homogenized at 10,000 r/min for 1 min to form an emulsion. Then, 100 μL of the sample was taken from the bottom at 0 and 10 min after homogenization, diluted with 10 mL of the 0.1% (*w*/*v*) SDS solution and the absorbance was measured with the TU-1810 UV-VIS spectrophotometer (Beijing Persee General Instrument Co., Ltd., China) at 500 nm, with the absorbance at 0 min. Indications of the emulsifying activity (EAI) and emulsifying stability (ES) were expressed by the emulsifying stability index (ESI) [14]. Each sample was measured three times, and the results were averaged.

(2) $$ESI=\frac{A_{0}\times ∆T}{∆A}$$

where A_0_ are the absorbance value at 0 min; ΔT is the time difference, which is 10 min in this test; ΔA is the absorbance value difference within.

#### 2.2.4. Determination of Foaming Properties

The determination of foaming power: the LWE sample was diluted to a protein solution with a protein content of 0.2% (*w*/*v*) with a phosphate-buffered solution (pH = 7.0, 0.1 mol/L). A total of 100 mL of diluent was taken, dispersed with a high-speed disperser at a speed of 10,000 r/min for 2 min, and a ruler was inserted to read the foam height [15]. The foaming force was calculated as follows:

(3) $$FC(\%)=\frac{V_{0}}{V}\times 100\%$$

where V_0_ is the volume of foam at 0 min after whipping; V is the initial liquid volume before whipping.

The determination of foam stability: the whipped sample was placed still for 30 min, and a ruler was inserted to read the foam height. The calculation method of foam stability is shown in the following formula:

(4) $$FS\left(\%\right)=\frac{∆V}{V_{0}}\times 100\%$$

where V_0_ is the volume of foam at 0 min after whipping; ΔV is the volume difference of foam before and after standing still.

#### 2.2.5. Determination of Free Sulfhydryl Content

The sulfhydryl content was determined according to Ellman’s reagent [16]. A total of 0.1 mol/L of the Tris-glycine buffer (pH 8.0, containing 0.01 mol/L EDTA) diluted 0.2 g of LWE to 10 mg/mL. A total of 1 mL of the diluent was removed, and 3 mL of the buffer solution was added and mixed well; a total of 40 μL of the DTNB reagent was added, kept warm in a water bath at 25 ± 1 °C for 30 ± 1 min, and the absorbance was measured at 412 nm. In the test, no sample was added, and the Ellman reagent was added as a blank; the turbidity was determined by adding the sample solution without the Ellman reagent. The sulfhydryl content was calculated according to the formula:

(5) $$SH(\mu \mathrm{mol}/\mathrm{mg})=75.53\;\times A_{412}\times D/C$$

In the formula: A_412_—the absorbance value of the sample when DTNB is added minus the absorbance value of the sample when DTNB is not added; C—the concentration of the egg liquid; D—the concentration of the sample.

#### 2.2.6. The Determination of Surface Hydrophobicity

The sample was diluted with 0.01 mol/L of the phosphate-buffered solution (pH = 7.0) to a protein concentration between 0.005–0.30 mg/mL (5 samples). A total of 4 mL of the diluted samples was taken with different concentrations; a Hitachi F2700 fluorescence spectrometer (Tokyo, Japan) was used to measure the fluorescence intensity (FI_0_) of the sample at the excitation wavelength of 395 nm and the emission wavelength of 475 nm, respectively, and the sample after adding 20 μL of the ANS solution (8 mol/L). For the fluorescence intensity (FI_1_), the difference between FI_1_ and FI_0_ is recorded as FI, the protein concentration is taken as the abscissa, and FI is used as the ordinate to draw a graph. The initial slope of the curve is the surface hydrophobicity index of the protein molecule, which is recorded as S_0_ [17].

#### 2.2.7. Sodium Dodecyl Sulphate–Polyacrylamide Gel Electrophoresis (SDS-PAGE)

The gel electrophoresis under reducing and non-reducing conditions were performed according to the method of Cui et al. [18]. A total of 40 µL of the protein solution (2 mg/mL) was added to 10 µL of the reducing loading buffer (5 Ⅹ) (Beyotime, Shanghai, China), and the other group was added to a non-reducing loading buffer (5 Ⅹ) and heated in a boiling water bath for 5 min. A total of 10 µL of the protein solution was loaded on the gels with a thickness of 1 mm composed of 12% separation gel concentration and 5% concentration gel and run at 120 mV. Molecular mass standards of 10.0–180.0 kDa (Biosharp, Beijing, China) were used to estimate the molecular mass of samples. Gels were scanned using a two-color infrared laser imaging system (Odyssey CLX, LICOR, Durham, NH, USA).

#### 2.2.8. Rheological Properties

The LWE samples were heated at 55, 58, 61, 64 and 67 °C for 4 min, respectively, and the rheological properties of the liquid whole egg were measured. For the dynamic rheological properties: Firstly, the stress scan determined the linear viscoelastic range of LWE, and then measured the storage modulus (G′) and energy modulus (G”) with the oscillation frequency. For the experimental conditions—plate spacing: 1 mm; temperature: 25 °C; oscillation frequency: 0.1–10 Hz; oscillation stress: 1%. For the determination of the viscosity of liquid whole egg, a MCR 302 rheometer (Anton Paar, Austria) was used to measure the change in the viscosity of LWE after different treatments, and the change in viscosity was measured with the shear rate to determine the rheological properties of LWE. For the experimental conditions: a 60 mm plate system; plate spacing: 1 mm; temperature: 25 °C; shear rate range: 1–100 s^−1^.

The Ostwald–de Waele model was used to analyze the viscoelastic properties of a sample. The model formula is:

(6) $$\eta =\frac{\tau }{\gamma }=k\times \gamma^{n-1}$$

where η is the viscosity (Pa), γ is the shear stress (Pa), τ is the shear rate (s^−1^), k is the consistency index (Pa s^n^), and n is the flow behavior index.

The power law function is often used to fit the frequency dependence of a sample, and therefore, the frequency dependence of G′ (G″) of the sample was fitted using the following power law function:

(7) $$G^{\prime }=k^{\prime }\times \omega^{n\prime }$$

(8) $$G^{\prime \prime }=k^{\prime \prime }\times \omega^{n\prime \prime }$$

where K′ (K″) and n′ (n″) represent the power law constants and frequency indices, respectively.

### 2.3. Statistical Analysis

Three parallel samples were made for each sample. Origin Pro 8.6 software was used for mapping and SPSS Statistics 19.0 software was used to analyze the experimental data. *p* < 0.05 indicated significant difference.

## 3. Results and Discussion

### 3.1. Appearance and Solubility

**Figure 1:**
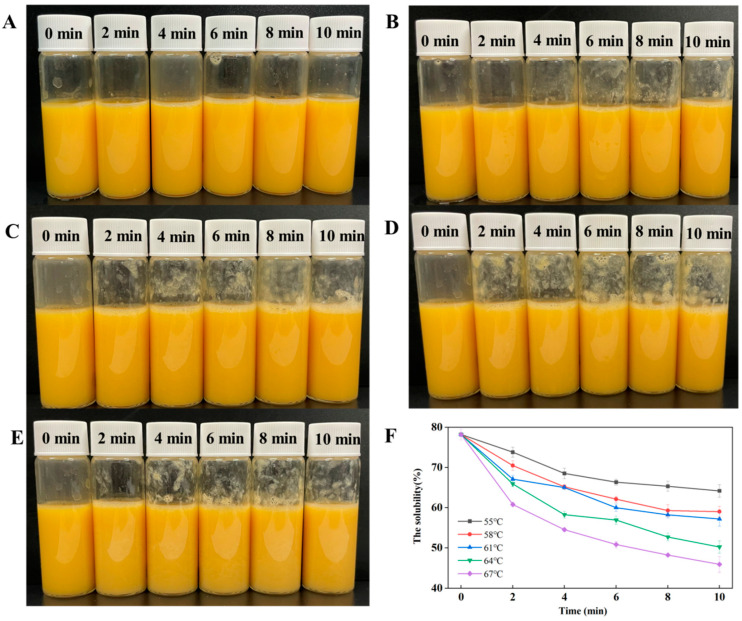
Effect of different heating treatment conditions on the appearance and solubility of LWE. (**A**): heating treatment at 55 °C; (**B**): heating treatment at 58 °C; (**C**): heating treatment at 61 °C; (**D**): heating treatment at 64 °C; (**E**): heating treatment at 67 °C; (**F**): solubility.

The heating treatments conditions could affect the appearance of LWE. As shown in Figure 1A–E, with the increasing heating temperature or times, the LWE appearance showed more flocculation than that of heating treatment at 55 °C (Figure 1A). It also can be seen from Figure 1F that heating treatment can significantly affect the solubility of LWE. When the heating time was constant, the protein solubility decreased by 17.94% (55 °C), 24.55% (58 °C), 26.89% (61 °C), 35.77% (64 °C) and 41.29% (64 °C), respectively, which shows a decreasing trend. When the heating temperature was constant, the solubility showed a downward trend with the extension of the heat treatment time. There were two main reasons causing the changes: (i) Heating treatment makes the protein partially denatured, the protein molecules expand, the hydrophobic groups are exposed, and the hydration of the protein is reduced. (ii) Unfolding protein molecules will form insoluble polymers through hydrophobic interaction, resulting in a decrease in the solubility of the liquid whole egg [1].

### 3.2. Emulsifying Properties

**Figure 2:**
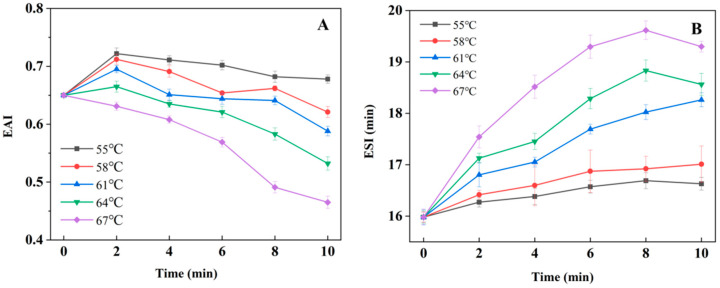
The effect of different heating treatment conditions on the emulsifying properties of LWE. (**A**): emulsifying activity (EAI); (**B**): emulsifying stability (ESI).

It can be seen from Figure 2A that after heating treatment at 55–64 °C, the emulsifying activity (EAI) of LWE generally increases first and then decreases with the extension of treatment time. The higher the temperature, the greater the decrease in the EAI of LWE. Under these temperature conditions, the EAI increased to different degrees when heating for 2 min, and the emulsifying activity increased by 11.07%, 9.54%, 6.92% and 1.50% under the temperature at 55, 58, 61 and 64 °C, respectively. This is because the emulsifying properties of proteins are related to surface hydrophobicity and molecular flexibility, and the protein emulsifying ability is weakly positively correlated with surface hydrophobicity. After heating treatment at 55–64 °C for 2 min, the LWE will lead to the partial denaturation of protein molecules, further expansion of molecular structure, increase in hydrophobicity on the surface of protein molecules, and increase in emulsifying activity. After heating treatment at 67 °C, the emulsifying activity of LWE was significantly reduced (*p* < 0.05). Under the condition of the heat treatment temperature at 67 °C, with the extension of treatment time, yolk high phosphoprotein and low-density lipoprotein and other proteins with their main role in emulsification gradually denatured, their solubility decreased, the proteins adsorbed to the oil–water interface were reduced, and the flexibility of proteins was reduced, resulting in a reduced emulsification performance [19,20,21].

As shown in Figure 2B, the emulsifying stability (ESI) of LWE does not change significantly under the condition of 55–58 °C (*p* > 0.05), while the ESI of liquid whole egg increases significantly after heat treatment at 61 and 67 °C (*p* < 0.05). The ESI of liquid whole egg increased by 14.28%, 16.15% and 20.75% after heat treatment at 61, 64 and 67 °C, respectively. It may be that the protein ESI is related to the hydrophobicity of the protein surface and the viscosity of the liquid egg. The heating treatment of the LWE at 61–67 °C will lead to the partial denaturation of the protein and increase in the hydrophobicity of the protein surface [21,22].

### 3.3. Foaming Activity

**Figure 3:**
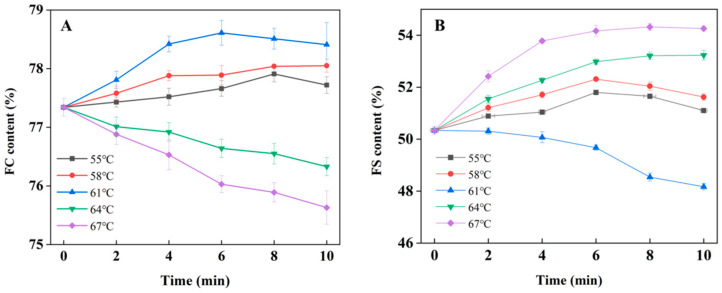
The effect of different heating treatment conditions on the foaming activity of LWE. (**A**): foaming activity (EAI); (**B**): foaming stability (ESI).

It can be seen from Figure 3A that in the range of 55–58 °C, the foaming activity of LWE gradually increases with the extension of the treatment time. At 61 °C, the foaming activity showed a trend of first increasing and then decreasing, which was better than that of the control group. The results showed that the appropriate heating treatment can effectively improve the foaming activity of LWE. The heating treatment is conducive to the formation of the water–air interface and the dissociation of the oomucin complex, which significantly improved the foaming activity of LWE. This is consistent with the findings of Plancken et al. [23]. When the heat treatment temperature was increasing to 64 and 67 °C, the foaming activity of liquid whole egg decreased significantly with the extension of treatment time, which indicates that the too-high heating temperature will reduce the foaming activity of LWE. This may be due to the increase in the amount of egg protein denaturation; the decrease in protein solubility; and the protein in the liquid egg existing in the form of polymers, which is not easy to expand and adsorb at the water–air interface, resulting in reduced foaming activity [24,25].

As shown in Figure 3B, compared with the control group, the foaming stability of LWE showed a trend of increasing and decreasing after heat treatment at 55 and 58 °C, reaching a maximum value when the heating treatment time was 6 min. This may due to the fact that the lysozyme–oomucin complex and oomucin in egg protein play a role in stabilizing foam, and that heating treatment at 55 and 58 °C can disintegrate the oomucin–protein complex and increase the stability of foaming. The foaming stability of LWE gradually decreased with the extension of the heating time at 61 °C. The foaming stability of LWE could be significantly improved by heating treatment at 64 and 67 °C, and the foaming stability increased with the extension of heating time. In addition, the viscosity of the medium can hinder the aggregation and coagulation of the dispersed phase and increase the foam stability, while 64 and 67 °C can significantly increase the foam stability of the LWE [26,27].

### 3.4. Surface Hydrophobicity and Sulfhydryl Group Content

**Figure 4:**
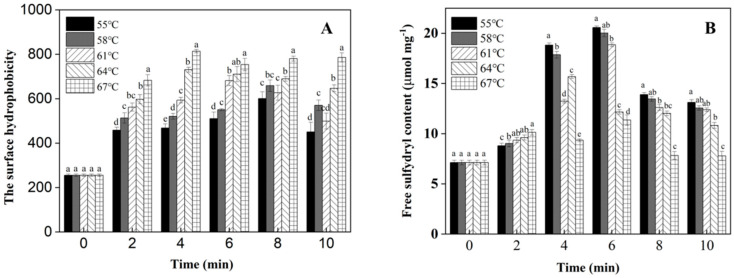
The effect of different heating treatment conditions on the surface hydrophobicity and sulfhydryl group content of LWE. (**A**): surface hydrophobicity; (**B**): sulfhydryl group content; ^a–d^ means within a row with different superscript letters indicate significant differences (*p* < 0.05). Mean results of 3 independent trials ± standard deviation.

Figure 4A shows the effects of heating temperature and heating time on the surface hydrophobicity of LWE. The surface hydrophobicity of the protein was significantly increased after heating treatment (*p* < 0.05). This is due to the partial denaturation of the protein, the unfolding of the protein molecular structure and the exposure of the hydrophobic groups buried inside the molecule, which increased the hydrophobicity of the protein [28]. The surface hydrophobicity of LWE reached its maximum value after heat treatment at 55 and 58 °C for 8 min, and the surface hydrophobicity decreased by extending the heating time. This is due to the fact that heating can make the protein structure unfold, the hydrophobic groups exposed and the surface hydrophobicity gradually increase, reaching the maximum value at 8 min. Meanwhile, the extended protein molecules will be aggregated into large molecules by the forces such as hydrophobic interactions, and the hydrophobic groups are encapsulated again, resulting in the decrease in protein hydrophobicity [19,29]. Similarly, the surface hydrophobicity content of the LWE reached the maximum at 6 min after heating treatment at 61 and 64 °C, respectively. After heating treatment at 67 °C, the surface hydrophobicity of LWE does not change significantly with the extension of heating time. An exceeding heating temperature caused the yolk proteins to be gradually denatured and the hydrophobic groups to be exposed; thus, the surface hydrophobicity of LWE does not decrease significantly.

Figure 4B shows the variation trend in sulfhydryl content on LWE with the heating temperature and heating time. The sulfhydryl content of the LWE changed significantly after heating treatment. The sulfhydryl content of LWE showed an overall trend of first increasing and then decreasing after heat treatment from 55 to 64 °C. The sulfhydryl content reached its maximum at 55, 58 and 61 °C when the heating time was 6 min, reached its maximum at 64 °C for 4 min, and decreased when the heating time continued to be extended. This may be due to the fact that heating treatment partially denatured the egg proteins, unfolded the protein molecules and exposed the sulfhydryl groups buried inside the protein molecules, which increased the protein sulfhydryl content [30]. When the heating time continued to extend, the exposed sulfhydryl groups were oxidized or cross-linked with each other to form -SH-SH-disulfide bonds under heating conditions, which decreased the sulfhydryl content of the proteins. The sulfhydryl content of egg proteins did not change significantly under the heating conditions at 67 °C, and the sulfhydryl content of egg proteins heated at this temperature was lower than that under other temperature conditions.

### 3.5. SDS-PAGE

**Figure 5:**
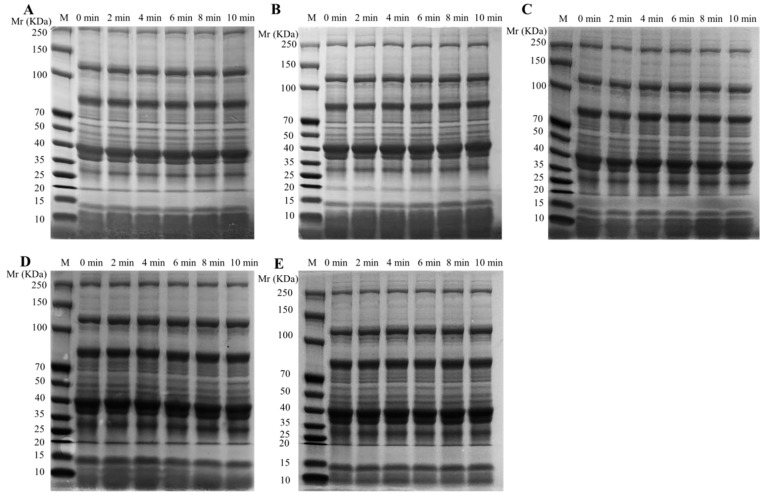
The effect of different heating treatment conditions on the SDS-PAGE of LWE. (**A**): heating treatment at 55 °C; (**B**): heating treatment at 58 °C; (**C**): heating treatment at 61 °C; (**D**): heating treatment at 64 °C; (**E**): heating treatment at 67 °C.

The LWE samples with different heating treatments were analyzed by SDS–PAGE (Figure 5), which showed molecular changes in the 10–250 kDa region. Previous studies suggested that the molecular masses of ovotransferrin, apoproteins of low-density lipoprotein (apo-LDL), ovalbumin and apoproteins of high-density lipoprotein (apo-HDL), ovomucoid, may exhibit molecular masses near 75 kDa, 50–70 kDa, 35–45 kDa, and 28 kDa, respectively [31,32]. The electrophoresis bands in the range of 28 kDa, 35–45 kDa and 50–70 kDa gradually deepened with the increase in temperature, which suggested that heating treatment significantly affected the content of apo-LDL, apo-HDL, ovomucoid and ovalbumin in LWE. This further indicated that the degree of protein denaturation gradually increased with the increase in temperature. This may be caused by protein denaturation and aggregation to form insoluble substances. Furthermore, the molecular weight of main bands in exterior egg yolks are near 15 kDa, and these bands are significantly weakened after heating treatment [31]. This showed that low molecular weight proteins form high molecular weight proteins through thermal polymerization. However, the effect of different heating treatment times on SDS-PAGE bands of LWE samples is not obvious. These results indicated that the change in protein abundance may be due to the polymerization by heating treatment, resulting in an increase in the abundance of high molecular weight protein and a decrease in the abundance of relatively small molecular weight protein.

### 3.6. Rheological Properties

**Figure 6:**
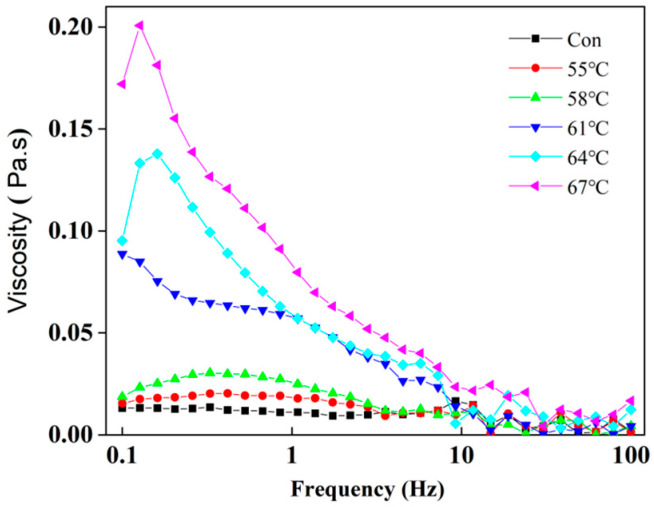
The ffect of different heating treatment conditions on the rheological properties of LWE. The “Con” in figure refers to control group.

The viscosity of LWE can affect the material transportation and product quality, and the study of the viscosity of egg liquid can provide a theoretical basis for the improvement of the process and equipment, which is of important guidance in food production. As can be seen from Figure 6, the higher the heating treatment temperature, the greater the viscosity of the LWE, which is due to the heating treatment that partially denatures the egg protein molecules, and the denatured protein molecules stretch out their structures and form large particles by intertwining between protein molecules, which increases the viscosity of the liquid whole egg. The viscosity of the fresh egg fluid in the blank group did not change significantly with increasing shear rate, indicating that the fresh egg fluid is a Newtonian fluid. The higher heating temperature, the more significant the shear thinning was, which indicated that the LWE became a pseudoplastic fluid after heating treatment, and changed the viscosity of the LWE [33]. 

Table 1 shows the values of the flow behavior index (n) and consistency index (k) determined by fitting the shear rate (τ) and apparent viscosity (η). The flow behavior index n indicates the degree to which a fluid differs from a Newtonian fluid. A fluid is considered a Newtonian fluid when n = 1, a pseudoplastic fluid (shear-thinning fluid) when 0 < n< 1, and a dilatant fluid when 1 < n< ∞. The results show that the rheological behavior index (n) ranges from 0.02660 to 0.03443, indicating that the heat-treated sample exhibits shear-thinning behavior within the range of shear rates (0.1–100 s^−1^), which is a typical rheological characteristic of non-Newtonian fluids. The consistency index (k) increases from 0.01093 to 0.05878, indicating that the apparent viscosity of whole egg gradually increases. This result is consistent with Souza’s findings, which showed that the apparent viscosity of egg increased gradually with the extension of the pasteurization time [34].

**Figure 7:**
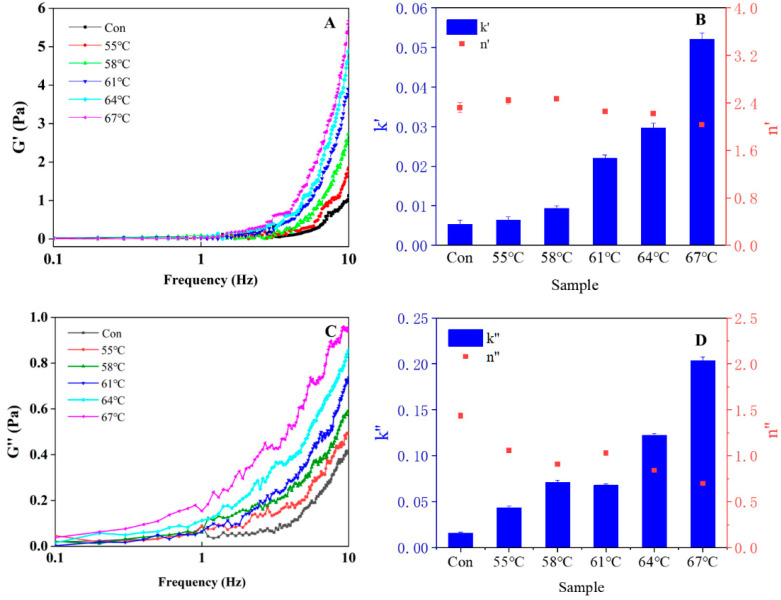
The effect of different heating treatment conditions on the frequency scanning diagram of LWE. (**A**): the storage modulus; (**B**): The Ostwald–de Waele model of the storage modulus; (**C**): the loss modulus; (**D**): The Ostwald–de Waele model of the loss modulus. The “Con” in figure refers to control group.

Figure 7A,C show the effect of heating treatment on the storage modulus (G′) and loss modulus (G″) of LWE. As can be seen from Figure 7A,C, the G′ and G″ of LWE increased with the increase in heating temperature. This indicates that heating treatment increases the viscoelasticity of the LWE. At the same time, the G′ and G″ of the LWE were significantly increased after heating treatment at 67 °C, which may due to the partial denaturation of egg protein [35].

The dependence of G′ and G″ on shear frequency can provide valuable information on the structure of egg yolk. The relationship between G′ and G″ and frequency was fitted using the power law model, and the regression parameters are shown in Figure 7B,D. By comparing the data in the figure, it can be observed that heat treatment causes a significant increase in K′ and K″ values, indicating that heat treatment enhances the cross-linking between protein molecules. The values of n′ and n″ of different treated samples show that there is no significant change in the frequency dependence of the viscoelasticity of whole egg after different heat treatments, indicating that the viscoelasticity of whole egg after different heat treatments is not significantly affected by the frequency dependence.

## 4. Conclusions

This study revealed the effect of heating treatment on the structure and functional properties of liquid whole egg (LWE). Indeed, the heating treatment provided liquid whole egg with functional properties similar to the raw treated samples, while the foaming capacity was even better. The results showed that the solubility of LWE decreased after heating treatment. The higher the temperature and the longer the time, the more the solubility of LWE decreased. After the proper heating treatment of LWE, the molecular structure expands, and the surface hydrophobicity of protein molecules increases, which is conducive to the play of functional properties such as emulsification, foam ability and the rheological properties of LWE. Heating treatment significantly affected the content of apo-LDL, apo-HDL, ovomucoid and ovalbumin in the LWE. The heating treatment at 61 °C for 6 min had the best effect on the LWE quality. However, exceeding the heating treatment reduces the functional properties of proteins, which thoroughly increases the denaturation of protein molecules, and the extended protein molecules intertwine into macromolecules. Therefore, the study can provide a theoretical basis for using the heating treatment to produce the liquid whole egg products.

## Figures and Tables

**Table 1:** The effect of different heating treatment conditions on the static rheological parameters of WEL.

Sample	K (pa·s^2^)	n
Con	0.01093	0.02884
55 °C	0.01458	0.02823
58 °C	0.01894	0.03443
61 °C	0.04444	0.02660
64 °C	0.05878	0.02920

## Data Availability

Data supporting the reported results are available upon request.
